# Affect labeling: The role of timing and intensity

**DOI:** 10.1371/journal.pone.0279303

**Published:** 2022-12-29

**Authors:** Einat Levy-Gigi, Simone Shamay-Tsoory

**Affiliations:** 1 Faculty of Education Bar-Ilan University, Ramat-Gan, Israel; 2 Gonda Multidisciplinary Brain Research Center, Bar-Ilan University, Ramat-Gan, Israel; 3 Department of Psychology, University of Haifa, Haifa, Mount Carmel, Haifa, Israel; Yamaguchi University: Yamaguchi Daigaku, JAPAN

## Abstract

A growing number of studies have shown that labeling negative feelings can down-regulate distress. The present study aimed to test the effectiveness of affect labeling while manipulating two factors known to influence the emotion regulation process, namely timing, and emotional intensity. In Experiment 1, sixty-three participants completed a performance-based affect labeling paradigm in which they had to choose between two labels that best describe their feeling. Participants were randomly assigned to one of three experimental conditions: (1) Simultaneous labeling- the labeling occurs while watching the aversive picture. (2) Subsequent labeling- the labeling occurs immediately after watching the aversive picture. (3) Delayed labeling- the labeling occurs 10 seconds after watching the aversive picture. We found that affect labeling efficiently down-regulated distress independent of the labeling timing. In Experiment 2, seventy-nine participants utilized simultaneous labeling for aversive pictures with low and high intensity. We revealed that while affect labeling reduces distress in high-intensity aversive conditions, it increases distress in low-intensity conditions. The results question the standard advice, which calls to count to 10 before you speak in highly aversive states. In addition, it suggests that affect labeling can be beneficial in high-intensity conditions. However, it should be used with caution in low-intensity conditions.

## Introduction

Affect labeling refers to the process of naming and describing our feelings [1]. Behavioral and neuroimaging studies suggest that merely putting feelings into words can serve as a regulatory strategy. Specifically, it diminishes emotional neural reactivity and helps minimize the adverse reaction to aversive or unpleasant stimuli [1–5]. While highly important, these studies have focused mostly on characterizing specific situations in which it is helpful (e.g., fear of spiders and public talking). Our study takes a step forward, trying to identify more general characteristics of this process. According to the process model of emotion regulation, two factors that substantially influence the effectiveness of the regulation process are timing and emotional intensity [6–8]. The present study aimed to characterize the efficacy of affect labeling in reducing distress while focusing on these two central factors in the emotion regulation process.

The process model of emotion regulation holds that individuals may regulate their emotions at five different points across the emotion-generative process: (a) selection of the situation, (b) modification of the situation, (c) deployment of attention, (d) change of cognitions, and (e) modulation of responses. According to the model, regulatory strategies applied during the first four stages are considered antecedent-focused strategies. In contrast, strategies applied during the response modulation stage are considered response-focused strategies. Moreover, the model suggests that emotions develop and gain strength across the emotion regulation process [9, 10]. Accordingly, since antecedent-focused strategies start operating early in the emotion-generative process, they are more effective when implemented close to the emotional stimuli before the emotional reactions are fully activated. On the other hand, response-focused strategies start operating after the emotion response tendencies are more fully activated and are more effective at a later stage of the emotion regulation process [11, 12].

While the process model of emotion regulation defines different regulatory strategies, including reappraisal, distraction, and suppression, it does not include references to affect labeling [13]. Affect labeling can be viewed as an antecedent-focused strategy since it may take place during the cognitive-change phase to describe the emotional stimuli [5]. For example, a researcher who gives a talk at a conference may use “excitement” or “stress” to describe their feelings during the talk. Moreover, like other antecedent-focused strategies, it requires a selection between different meanings attached to the situation [14]. Finally, it involves the exact brain mechanisms as reappraisal, a common antecedent-focused strategy [15–17]. According to this view, and in line with the process model of emotion regulation, it would be more effective at an early stage of emotional processing before the negative emotion becomes more intense [11, 12].

However, similar to response-focused strategies such as suppression [10], affect labeling may reflect a tendency to influence the response after the emotion is developed. For example, individuals with a fear of spiders may use labels such as “fear” or “anxiety” to describe their feelings after encountering one. Hence, it can be viewed as a response-focused strategy. According to this approach, it will be most effective at the later stages of the emotion regulation process.

One way to examine the antecedent vs. response-focused nature of affect labeling is to test whether its effectiveness changes as a function of the regulatory timing. Most studies investigating affect labeling have used labeling close to the emotional stimuli [e.g., 1, 4, 16, 18, 19, for a review see 5]. However, it is not yet clear whether the effectiveness changes over time. The aim of Experiment 1 was to test the interplay between the timing of labeling and its effectiveness in reducing distress. To that end, we compared the effectiveness of affect labeling when applied simultaneously, subsequently, or 10-seconds after the aversive stimuli. The different intervals were chosen based on previous studies which used EEG to measure brain activation or heart rate to assess changes in physical arousal following watching aversive pictures from the International Affective Picture System (IAPS) [20]. These studies suggest that different regulatory strategies are activated at different times, starting at the appearance of the emotional stimulus, and ending approximately after 10 seconds [21–23].

Another factor that affects the effectiveness of affect labeling is the intensity of the regulated stimuli [11, 12]. Studies revealed that in high-intensity conditions, individuals prefer to apply disengagement and less demanding regulatory strategies, such as distraction, rather than more engaging strategies, such as reappraisal [24, 25]. On the one hand, affect labeling may be viewed as an engagement strategy that aims to provide meaning to a given stimulus. On the other hand, it has unique characteristics, which may question such an approach; first, common meaning-making strategies are more effortful since they involve more conscious and deliberate efforts, whereas affect labeling involves mainly reflecting and verbalizing one’s emotional state [16]. In addition, while meaning-making strategies aim to reduce negative feelings by changing the meaning of the stimuli to be more neutral, affect labeling aims to simply define and specify the negative emotion with no conscious intention to modify the emotional response [26]. Therefore, it can be viewed as a less demanding process.

The less demanding nature of affect labeling, together with its simple aim to define rather than change the aversive condition, may suggest that it would be highly effective in high-intensity situations. Indeed, a review of the literature on affect labeling reveals that most studies have used it to reduce distress in highly extreme conditions, including public speaking tasks [19], exposure to spiders [3], or when watching highly negative emotionally arousing pictures from the International Affective Picture System (IAPS) [2, 16, 18, 20]. However, it is unclear whether it is effective, mostly in high-intensity conditions or also in low-intensity conditions. Building on studies showing that venting may increase emotional intensity [27, 28], one possibility is that affect labeling would increase emotional intensity in low-intensity conditions. To answer this question, Experiment 2 aimed to compare the effectiveness of affect labeling in aversive conditions with low and high intensities.

## Experiment 1

This experiment aimed to test whether the timing of affect labeling would influence its effectiveness in reducing distress. Based on studies that demonstrated the regulative value of affect labeling [1, 2, 5, 29], we predicted that levels of distress in the affect labeling trials would be lower compared to baseline (as measured in the passive viewing trials). Since the nature of affect labeling is unclear [13], we did not have a specific hypothesis on the interplay between effectiveness and timing. Moreover, although a simple manipulation such as a 10-second delay cannot clearly distinguish between antecedent and response-focused strategies, it may provide initial evidence regarding the effects of labeling timing. Suppose affect labeling would be most effective when implemented close to the emotional stimuli. In that case, it may be more closely related to other antecedent-focused strategies. However, if it would be primarily effective when implemented after a 10- second delay, it would be more closely associated with other response-focused strategies.

### Participants

The sample size was calculated using the G*Power software [30]. Based on an effect size that was found in a previous study [31], we conducted a-priori power analysis for repeated measures ANOVA based on the ability to detect a medium-size effect (Cohen’s f = .27), with a significance level (α) of 5% and power level (1-β) of 95% [32]. The analysis revealed the need for 57 participants for the current study. We increased the number by 10% to account for possible equipment failure and participant drops. Sixty-three individuals participated in this study (See Table 1 for a detailed sample description). Participants were recruited using advertisements on the campus and surrounding area. Exclusion criteria for all participants were assessed using the MINI international psychiatric interview [33]. They included current or past diagnosis of psychiatric disorders, risk of suicidal/homicidal ideation, any substance dependence or abuse within the past six months, a history of concussion or other clinically significant head injuries, including loss of consciousness for over ten minutes or a history of neurological disorders such as epilepsy, multiple sclerosis, stroke or encephalitis. None of the participants was excluded from this experiment. The following report includes all measures and conditions used in the study. All the participants signed a written consent form prior to the beginning of the study. The university ethics board approved the study (approval number 010/16).

**Table 1:** Demographic characteristics of individuals who participated in Experiments 1 and 2 (means and standard deviations/frequency).

	Experiment 1 Mean (SD)	Experiment 2 Mean (SD)
Age (years)	30.25 (9.91)	29.18 (9.21)
Female/male (Ns)	(42/21)	(52/27)
Education (years)	14.30 (2.45)	14.51 (2.21)
Depression	6.73 (7.74)	7.43 (8.46)
Anxiety	63.59 (20.24)	70.06 (18.74)
Difficulty to describe feelings	15.62 (5.30)	16.35 (5.32)

### Measures and procedure

#### The affect-labeling paradigm

**Fig 1:**
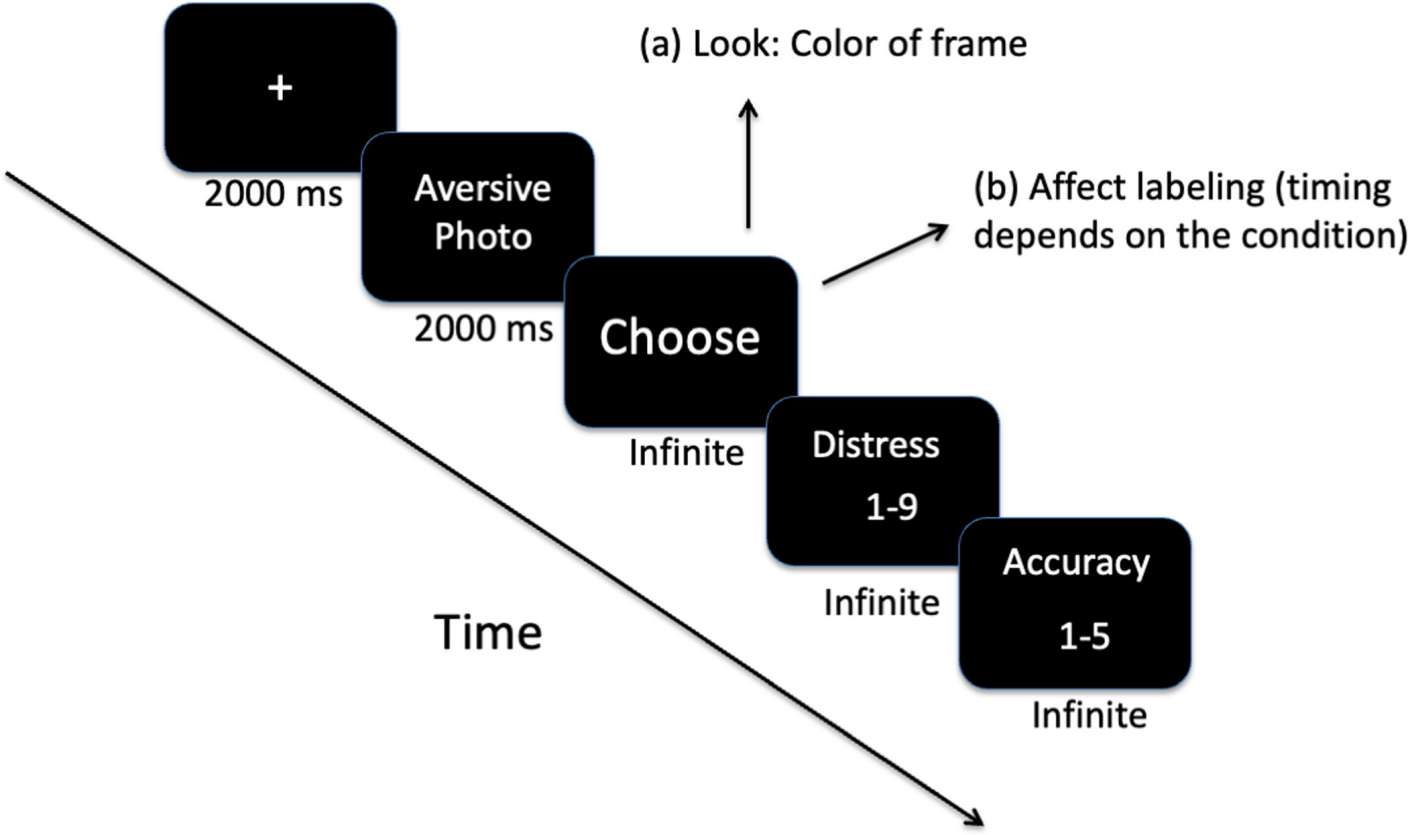
Illustration of the affect labeling task.

The participants completed an affect-labeling paradigm [34]. In this paradigm, we used pictures from the IAPS [20], which are ranked on a 1–9 Likert scale on arousal (1-low, 9- high) and valance (1-most negative, 9- most positive). We selected 60 pictures that were highly emotionally arousing (mean arousal = 6.42; mean valence = 1.78). Each image was presented twice, once to measure levels of distress when applying affect labeling and once as part of the control trials that measured baseline distress during passive viewing when no emotion regulation was applied. The order of the presentation was counterbalanced. For a detailed description of the procedure, see the S1 File. In all trials, the participants were asked to rate their level of distress immediately after their label/frame choice on a 9-point Likert scale (with higher numbers indicating more significant distress) and show how accurately the selected label describes their feelings on a 5-point Likert scale (with higher numbers indicating greater accuracy). See Fig 1 for an illustration of the task. The participants in both experiments reported high levels of accuracy (*M* = 4.72, *SD* = .15), confirming that they selected labels that accurately described their feelings, with no significant differences in accuracy level as a function of intensity (*p* > .1).

To test whether the timing of labeling affects levels of experienced- distress, participants were randomly assigned to one of three experimental conditions as follows: (1) Simultaneous labeling- the labeling occurs while watching the aversive picture (N = 20). (2) Subsequent labeling- the labeling occurs immediately after watching the aversive picture (N = 21). (3) Delayed labeling- the labeling occurs 10 seconds after watching the aversive picture (N = 22). In each condition, color frame choices were delayed by the same interval. After completing the paradigm, participants were debriefed.

#### Self–report questionnaires

Some evidence suggests that anxiety, depression, and the ability to describe feelings may impact the effectiveness of affect labeling. Specifically, it was found that individuals with more significant public speaking anxiety benefited more from affect labeling compared to non-anxious individuals [19] and that affect- labeling predicts treatment responsiveness in both anxious and depressed individuals [35, 36]. Lastly, studies revealed that individuals with alexithymia have impaired affect labeling, and they fail to describe and identify emotional situations appropriately [13, 37–41]. To control for these possible effects, participants completed: (1) The State-Trait Anxiety Inventory [42] (α = .86); (2) The Beck Depression Inventory (BDI-II) [43] (α = .88); and (3) The Toronto Alexithymia Scale (TAS-20) [44]. Report from this scale refers only to the difficulty in describing feeling subscale (α = .86).

### Data analysis

We used SPSS (version 27) software (SPSS Inc., IL, USA) to analyze the data. A Kolmogorov–Smirnov test indicated that level of distress after affect labeling followed a normal distribution pattern (*D*(63) = .10, *p* = .20).

### Results and discussion

#### Zero-order correlations

Table 2 depicts zero-order correlations between levels of distress in control and affect labeling trials and depression, anxiety, and difficulty describing feelings. Note that the results in the three-timing conditions were redundant; hence, we collapsed them to simplify our report. In line with the existing literature [45, 46], there were significant positive correlations between anxiety and levels of both depression symptoms and difficulty in describing feelings. Notably, there were no significant correlations between levels of distress in the affect labeling and control trials and levels of anxiety, depression, and difficulty in describing feelings.

**Table 2:** Zero-order correlations between levels of distress in the different experimental conditions (baseline and affect-labeling), depression, anxiety, and difficulty in describing feelings.

	Baseline Distress	Affect Labeling	Depression	Anxiety	Describe feelings
Baseline Distress	1				
Affect Labeling	.85***	1			
Depression	.05	-.01	1		
Anxiety	.03	.001	.58***	1	
Describe feelings	.02	-.04	.19	.33**	1

** p < .005;

*** p < 0.001

#### Timing and the effectiveness of affect labeling

To test the effect of labeling timing on levels of distress, we conducted a regulatory strategy (affect labeling vs. control) X timing (simultaneous, subsequent, and delayed) repeated measures ANOVA analysis, with timing as a between-participant variable and regulatory strategy as a within-participant measure. As predicted, there was a significant main effect of strategy (*F*(1,60) = 13.20, *p* < .001, η^2^_p_ = .18), indicating that in line with previous studies when applying affect labeling, levels of distress become significantly lower compared to baseline. However, in contrast to our prediction, there was no significant main effect of timing (*F*(2,60) = .26, *p =* .97) and no significant timing by strategy interaction (*F*(2,60) = .03, *p* > .05), indicating that the timing of labeling does not influence the effectiveness of this strategy. Hence, affect labeling was equally effective whether it was applied simultaneously to the stimuli, subsequently after its presentation, or in a 10- second delay. In line with these results, in our second experiment, we kept the labeling timing as a constant, simultaneously with the aversive stimuli, while manipulating the intensity of these stimuli.

## Experiment 2

According to the process model of emotion regulation, another crucial factor in regulating distress is the intensity of the regulated situation. The goal of Experiment 2 was to test whether the effectiveness of affect labeling would change as a function of the intensity of the aversive stimuli. We suggest that affect labeling is less demanding than other meaning-making strategies; hence, it is easily applied and can be helpful in high-intensity conditions [25]. Indeed, it effectively regulates distress in highly aversive states [for review, see 5]. Since these conditions provoke extended levels of distress compared to low-intensity conditions, a broader range of improvement is possible. Taken together, we predicted that affect labeling would be more effective in high compared to low-intensity conditions. Since the labeling timing was found to have null effects, in this study all

### Participants

The sample size was calculated using the G*Power software [30]. Based on the effect size that was found in a previous study [31], we conducted a-priori power analyses both for the repeated measures ANOVA and for the process moderating model, based on the ability to detect a medium-large size effect (Cohen’s f = .24), with a significance level (α) of 5% and power level (1-β) of 95% (Cohen, 1992). The analysis revealed the need for 59 participants for the between-participant analysis [32] and a minimum of 75 participants for the cross-sectional moderating model analysis. Therefore, we recruited 82 participants to ensure sufficient power. The recruitment approach and the exclusion criteria were the same as in Experiment 1. Two participants were excluded due to technical problems. One participant chose to quit before completing the task. Hence 79 participants remained in the study (See Table 1 for a detailed description of the sample). The following report includes all measures and conditions used in the study. All the participants signed a written consent form prior to the beginning of the study. All the participants signed a written consent form prior to the beginning of the study.

### Measures and procedure

#### The affect labeling paradigm- low and high intensity

The participants completed an affect labeling paradigm similar to the paradigm used in Experiment 1. However, instead of using only highly aversive pictures we used pictures with low negative intensity (Arousal: Mean = 5.02, SD = .68, Range 3.95–5.87; Valence: Mean = 3.34, SD = .27, Range 2.9–3.73) or high negative intensity (Arousal: Mean = 6.15, SD = .67 Range 5.25–7.26; Valence Mean = 1.99, SD = .33, Range 1.45–2.59). Low and high intensities were defined in a similar way as in a large number of studies, based on well-validated criteria and were significantly different in their valence and arousal rating [see also 24, 25, 47].

### Data analysis

We used SPSS (version 27) software (SPSS Inc., IL, USA) to analyze the data. A Kolmogorov–Smirnov test indicated that the level of distress after affect labeling does not follow a normal distribution (*D*(79) = .11, *p* = .03).

### Results and discussion

#### Zero-order correlations

Table 3 depicts zero-order correlations between levels of distress in the four experimental conditions (baseline distress in conditions of low/high-intensity pictures, distress following affect-labeling in conditions of low/high-intensity pictures) and depression, anxiety, and difficulty in describing feelings. The general pattern of results is similar to the findings in Experiment 1. Specifically, we found no significant associations between levels of anxiety, depression, and alexithymia symptoms and the effectiveness of affect labeling in low and high-intensity conditions.

**Table 3:** Zero-order correlations between levels of distress in the different experimental conditions (baseline and affect-labeling), depression, anxiety, and difficulty in describing feelings.

	Baseline low	Baseline high	Labeling low	Labeling high	Depression	Anxiety	Describe feelings
Baseline low	1						
Baseline high	.68***	1					
Labeling low	.63***	.68***	1				
Labeling high	.59***	.86***	.80***	1			
Depression	-.17	.03	-.01	.00	1		
Anxiety	-.01	-.02	.00	.01	.58***	1	
Describe feelings	-.07	-.17	-.16	-.13	.31**	.37**	1

p < 0.05;

** p < .005;

*** p < 0.001

#### Affect labeling, negative intensity, and distress

**Fig 2:**
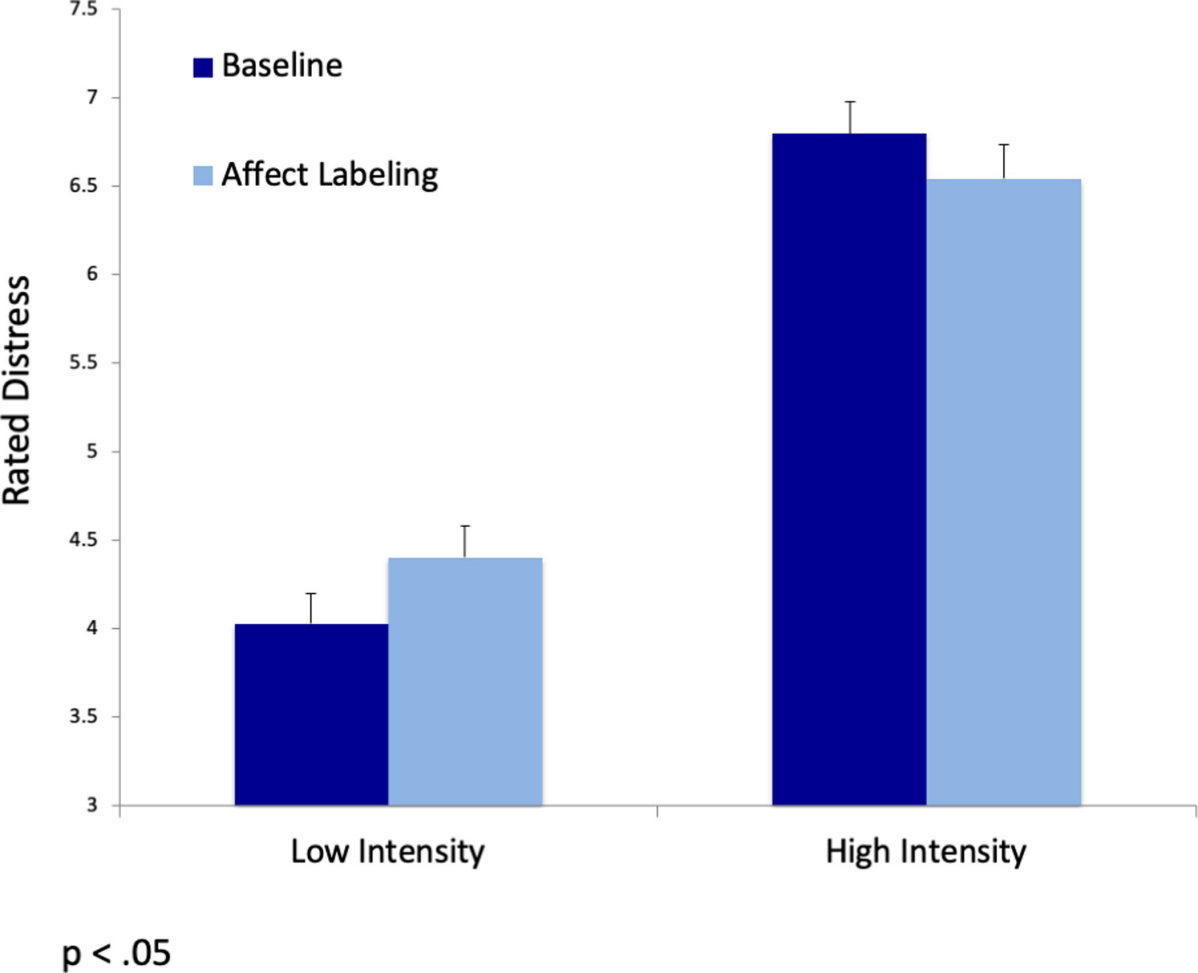
Level of distress (means and SE) as a function of regulation type (baseline/affect labeling) and intensity (low/high).

To examine our main prediction regarding the influence of negative intensity on levels of experienced distress, we conducted a strategy (affect labeling vs. control) X negative intensity (low vs. high) repeated measures ANOVA analysis, with regulatory type and negative intensity as within-participant measures. We found a significant interaction between strategy and intensity (*F*(1,76) = 22.37, *p* < .001, η^2^_p_ = .23). Follow-up paired-sample t-test analyses with Bonferroni correction (α = .025) revealed that while in low intensity conditions, affect labeling increased distress compared to baseline (*t*(78) = -2.6, *p* = .011), in high intensity conditions affect labeling decreased distress compared to baseline (*t*(78) = 2.4, *p* = .018). Please note that the current effect sizes are similar to those found in other related studies [1, 4, 34]. The results indicate that while affect labeling may help reduce distress in highly aversive conditions, it actually increases distress compared to baseline in low aversive conditions. The results are depicted in Fig 2.

## General discussion

The present study is the first study to characterize the conditions by which affect labeling is effective while focusing on two central aspects in the emotion regulation process, namely, timing and intensity. In contrast to our prediction, we found no significant effect of labeling timing. Hence, participants exhibited similar levels of distress whether the labeling co-occurred with the presentation of the emotional stimulus, subsequently to it, or after a 10- second delay. In terms of the process model of emotion regulation [6, 11, 12], these findings may suggest that unlike common antecedent-focused strategies such as reappraisal that are most effective in reducing distress when applied earlier in the emotion regulation process, affect labeling effectively reduce distress even at later stages of the emotional process after the negative emotion supposedly gained strength and not only close to it. A possible explanation for this difference is that antecedent-focused strategies, such as reappraisal, require the regulator to reframe a given situation (e.g., when watching two women hug and cry, thinking about how they support each other instead of the adverse circumstances which led to this moment). After the situation was already framed, an attempt to reframe it may result in competition between the old and new frames, and the shift might be more challenging. On the other hand, affect labeling requires no shifts nor manipulations but simply naming the feeling. Although the labels may change over time (e.g., something that was initially considered sad may later be viewed as hopeful), the effectiveness of the regulatory process solely refers to the naming itself and not to its accuracy of it [4, 34].

Testing the effectiveness of affect labeling as a function of stimuli intensity revealed that, as predicted and in line with previous studies, affect labeling was beneficial and reduced distress in high-intensity conditions [e.g., 1, 2, 4; for review see 5]. Nonetheless, in low-intensity conditions, not only was affect labeling less effective, but it actually increased levels of distress. Specifically, after watching low-intensity pictures, participants reported more significant distress when labeling their negative feelings compared to baseline. Notably, a higher level of distress in low-intensity conditions was obtained even though the participants agreed with the labeling and experienced it as a good description of their negative feelings.

One possible explanation for these results may relate to the priming effect caused by the labeling. Studies on the priming effect reveal that a response to a given stimulus is faster when related priming precedes it. Specifically, the priming activates a semantically associated network, which increases the availability of semantically related stimuli and hence decreases the reaction time to the given stimulus [48]. In the current study, the emotional label may serve as semantic priming, which leads to a spread activation of other related negative feelings [For review see 49]. These results align with the language as context view, which suggests that language shapes the processing involved in seeing emotion in another person’s face [50]. In high-intensity conditions, this activation corresponds with the solid and deep feelings that the presented stimuli initiate.

On the other hand, in low-intensity conditions, the presented stimuli may not activate such an intense negative network. However, labeling the negative feeling (as opposed to only viewing it passively), using one of the two suggested solid emotions may solely activate an intense associative network. This network may achieve the opposite aim. Hence, instead of down-regulating the negative feelings, it may up-regulate them and increase the levels of experienced distress.

In line with this view, it is possible that labeling has a dual effect; on the one hand, it increases emotion and does not allow it to disappear. On the other hand, it may enhance the awareness of emotional distress. Therefore, when the distress is mild, it does not allow returning to a homeostasis state. When the distress is high, it helps to return to a homeostasis state. Possible support for this view can be found in studies that showed that expressing some emotions, such as anger, is harmful. Hence, venting anger actually increases aggressive feelings [28] and adds fuel to the flame by heightening the activation of angry thoughts and action tendencies [27]. However, future studies which test homeostasis during affect labeling of low and high-intensity stimuli are needed to understand further the interplay between the effectiveness of affect labeling and the intensity of the situation.

Elevated levels of distress following affect labeling in low-intensity conditions may also be viewed in daily life. For example, drivers who verbally name their feelings about any annoying situation on the road may feel more distressed than those who passively view it. However, future research with naturalistic environments is needed to reach more conclusive results regarding these situations.

There were no correlations between the effectiveness of affect labeling in different timings or intensities and difficulty in describing feelings, depression, and anxiety symptoms. While this is only a first step toward understanding the relationship between the effectiveness of affect labeling and a limited number of symptoms, it may suggest that the ability to benefit from it is not limited to non-symptomatic individuals. However, it is essential to note that we used exclusion criteria in both experiments to determine the investigation only to subclinical symptoms that may be common in the general population. Future studies may further test individual differences by using larger samples and/or focusing on individuals with specific psychopathologies.

The results of the present study may have important clinical implications, especially considering the growing use of affect labeling as a complementary intervention method to reduce anxiety [3, 18, 19, 36, 51, 52]. Specifically, it is possible that the mere opportunity to choose between different emotional labels to describe feelings may serve as a simple and easy-to-apply approach that helps individuals to reduce distress in highly aversive situations. This might be particularly useful because our results suggest a more flexible application of affect labeling, allowing different intervals between the aversive situation and the labeling process. However, affect labeling should be used cautiously in low-intensity conditions since it may increase rather than decrease distress. Alternatively, affect labeling can be used as an intervention that aims to improve individuals with blunted affect to identify and describe various feelings and, hence, reduce apathy and differentiate between emotions with different intensities. Future research may further test the effect of intensity and timing in clinical populations, including individuals with blunted affect, alexithymia, anxiety disorders, hypervigilance, and different phobias, as part of basic and intervention-related studies to support and extend our conclusions.

The current study has several limitations. First, it was conducted in a lab setting, which is not necessarily equivalent to a real-world environment, and applied only behavioral measures. While this might be considered a significant limitation, it should be noted that previous studies on affect labeling revealed similar results whether conducted in the lab where participants had to regulate negative emotions in response to aversive pictures [e.g., 4], or in a field setting where participants had to cope with live spiders [3] or speak in public [19]. Furthermore, one of the main advantages of the current study is using a well-validated computer-based paradigm, which was modified to examine the issue of timing and intensity. However, along with the apparent advantages in precision, controllability, and measurability, computerized tasks create a highly artificial context devoid of the richness of real-life situations. Therefore, future studies may aim to expand the investigation to include real-life situations, manipulate the situation’s complexity, and apply additional physiological and neural measures. Such studies may contribute to a further understanding of the relationship between intensity and effectiveness and may shed light on the main related mechanisms that may improve the healing effect of affect labeling.

Second, the manipulation included only a 10- second delay. Evidence suggests significant differences in regulatory-related brain activation and physical arousal [21–23]. However, even such a delay is still relatively short in the scope of the real world, and, in some situations, labeling may take much longer. For example, labeling our emotions during a tense argument may take place only hours later when discussing it with a significant other, whereas recounting a traumatic childhood event may lead to affect labeling only after years. Future studies may aim to apply longer time intervals to further understand the effect of timing and its possible consequences for regulating emotions in daily life.

Another related limitation is that the participants had to choose between only two emotions to label their feelings in each situation. Whereas, in real- life, people generate these labels by themselves. However, such a design enables us to control for possible individual differences in generating feelings in aversive conditions. Moreover, a review of the literature on affect labeling [5] suggests that providing labels simplifies the regulatory process by reducing the choice space, making the labels more accessible, and reducing the need for introspection. Providing labels may be especially important when using affect labeling as a possible intervention. Specifically, it allows reaching relevant conclusions for treatment and educational settings, in which other people (e.g., the therapist or the parent) can propose different labels to facilitate emotion regulation and reduce levels of distress. Future studies may use naturalistic affect labeling, in which individuals generate their own emotions, to better imitate real-life situations.

Similarly, it is possible that although the labels were carefully chosen [see also 34], they did not capture the exact feelings of the participants. However, the participants in both experiments confirmed that they selected labels that accurately described their feelings. Future studies may test whether providing more emotional labels in each situation affects the results.

Finally, like other studies in the field, we tested psychiatrically healthy individuals while looking at the effect of the continuous level of symptoms rather than comparing those with and without a clinical diagnosis. Evaluating these individuals is essential as the first step towards a comprehensive understanding of affect labeling. Moreover, in line with the current research domain criteria (RDoC), it allows testing affect labeling on a continuous range of sub-clinical symptoms while focusing on the general mechanisms rather than a dichotomy [53, 54]. However, it is possible that clinically diagnosed individuals would show different result patterns. Specifically, they may perceive the stressors as more intimidating and experience a more significant level of distress. Further investigations may aim to understand affect labeling while focusing on clinically diagnosed populations. Such research would illuminate the role of affect labeling in different psychopathologies and its possible interaction with other cognitive and emotional dysfunctions as well as with given situational demands.

In conclusion, the present study provides essential insights into the nature of affect labeling. As a simple and straightforward tool that can easily manage stress and reduce distress in highly aversive conditions, independent of the labeling timing. However, it should be used with caution in low-intensity conditions since it may increase the level of experienced distress.

## Supporting information

S1 File A detailed description of the task and measures. (DOCX)
