# Supplementary material for: Affect labeling: The role of timing and intensity
Source: PLoS One. 2022 Dec 29;17(12):e0279303. doi: 10.1371/journal.pone.0279303 (PMC9799301; doi:10.1371/journal.pone.0279303)
Supplement: S1 File — (DOCX) [file pone.0279303.s001.docx]

**Supplementary Materials:**

**A detailed description of the task and measures**

A detailed description of the methods and procedure

**Experiment 1**

The order of the presentation was counterbalanced. Hence, for half of the images, the participants first used affect labeling (group 1), and for half, they first used passive viewing (group 2). The presentation order of the images was randomized across participants. The presentation duration was 2000 ms. In the affect labeling trials, participants had to choose between two negative emotional labels that appeared on the screen (for example, fear or disgust) a label that best describes their emotions. In the control trials, participants had to choose between two different frame colors to measure baseline distress when no emotion regulation was applied. We conducted a preliminary study to choose adequate emotional labels for each image. In this study, three independent evaluators, blind to the study hypothesis, viewed a set of 80 aversive images. They were asked to write down their two most recognizable feelings for each image. Based on this labeling procedure, we selected 60 images in which all three judges selected exactly the same labels (see also Shamay-Tsoory & Levy-Gigi, 2021).

In the experiment, participants indicated their choice by pressing the keyboard right (the letter Z) or left (the letter M). Frames were displayed around the images and labels below them. The location of the different emotions/frames’ colors (right or left side of the screen) was counterbalanced across trials. The effect of timing was tested in a between-subject design to avoid multiple presentations of each image, which may result in habituation and decrease.

**Experiment 2**

We conducted t-tests which showed significant differences between the low and high intensity sets (*t*(58) = -6.53, *p* < .001; *t*(58) = 17.69, *p* < .001 for arousal and valance, respectively). The general content and complexity of the low and high-intensity images were roughly matched (See Figure 1 for an illustration of low and high-intensity pictures taken from the open repository). In addition, there were no significant differences in the emotion categories across the low and high-intensity conditions. Since Experiment 1 revealed no significant differences as a function of timing, in Experiment 2, affect labeling occurred simultaneously with the aversive image in all the experimental conditions. The self-report questionnaires were similar to those we used in Experiment 1; internal consistency was .87; .84; .86 for the STAI, BDI, and difficulty in describing feelings, respectively.

**IAPS related details**

The IAPS numbers of the high intensity images that were used in Experiments 1 and 2 (N=60): 102, 105, 107, 112, 121, 123, 126, 127, 231, 232, 236, 238, 239, 240, 242, 243, 244, 245, 247, 248, 250, 1111, 2730, 2981, 3000, 3005, 3010, 3015, 3030, 3051, 3053, 3060, 3062, 3063, 3064, 3068, 3069, 3071, 3100, 3101, 3102, 3110, 3120, 3130, 3140, 3150, 3168, 3170, 3261, 3266, 3301, 3550, 6212, 6555, 9252, 9301, 9400, 9405, 9420, 9433.

The IAPS numbers of the high intensity images that were used in Experiment 2 (N=30): 1110, 1275, 1301, 2130, 2205, 2278, 2312, 2457, 2490, 2691, 2700, 2722, 2753, 6010, 6190, 6211, 6836, 6840, 7360, 9102, 9120, 9160, 9190, 9230, 9403, 9429, 9440, 9445, 9470, 9471.

It should be mentioned that all the low and high-intensity images used are high-validated and used in several other studies that used intensity (e.g., Levy-Gigi et al., 2016; Sheppes et al., 2011, 2014; Shafir et al., 2015).
